# Diastolic time – frequency relation in the stress echo lab: filling timing and flow at different heart rates

**DOI:** 10.1186/1476-7120-6-15

**Published:** 2008-04-21

**Authors:** Tonino Bombardini, Vincenzo Gemignani, Elisabetta Bianchini, Lucia Venneri, Christina Petersen, Emilio Pasanisi, Lorenza Pratali, David Alonso-Rodriguez, Mascia Pianelli, Francesco Faita, Massimo Giannoni, Giorgio Arpesella, Eugenio Picano

**Affiliations:** 1 Department of Echocardiography, Institute of Clinical Physiology, National Council of Research, Pisa, Italy; 2 Department of Digital Signal Processing Lab, Institute of Clinical Physiology, National Council of Research, Pisa, Italy; 3 Department of Cardiology, Hospital de Leon, Leon, Spain; 4 Department of Surgery and Transplants, University of Bologna, Italy

## Abstract

A cutaneous force-frequency relation recording system based on first heart sound amplitude vibrations has been recently validated. Second heart sound can be simultaneously recorded in order to quantify both systole and diastole duration.

**Aims:**

1- To assess the feasibility and extra-value of operator-independent, force sensor-based, diastolic time recording during stress.

**Methods:**

We enrolled 161 patients referred for stress echocardiography (exercise 115, dipyridamole 40, pacing 6 patients).

The sensor was fastened in the precordial region by a standard ECG electrode. The acceleration signal was converted into digital and recorded together with ECG signal.

Both systolic and diastolic times were acquired continuously during stress and were displayed by plotting times vs. heart rate. Diastolic filling rate was calculated as echo-measured mitral filling volume/sensor-monitored diastolic time.

**Results:**

Diastolic time decreased during stress more markedly than systolic time. At peak stress 62 of the 161 pts showed reversal of the systolic/diastolic ratio with the duration of systole longer than diastole. In the exercise group, at 100 bpm HR, systolic/diastolic time ratio was lower in the 17 controls (0.74 ± 0.12) than in patients (0.86 ± 0.10, p < 0.05 vs. controls).

Diastolic filling rate increased from 101 ± 36 (rest) to 219 ± 92 ml/m^2^* s^-1 ^at peak stress (p < 0.5 vs. rest).

**Conclusion:**

Cardiological systolic and diastolic duration can be monitored during stress by using an acceleration force sensor. Simultaneous calculation of stroke volume allows monitoring diastolic filling rate.

Stress-induced "systolic-diastolic mismatch" can be easily quantified and is associated to several cardiac diseases, possibly expanding the spectrum of information obtainable during stress.

## Introduction

**Figure 1:**
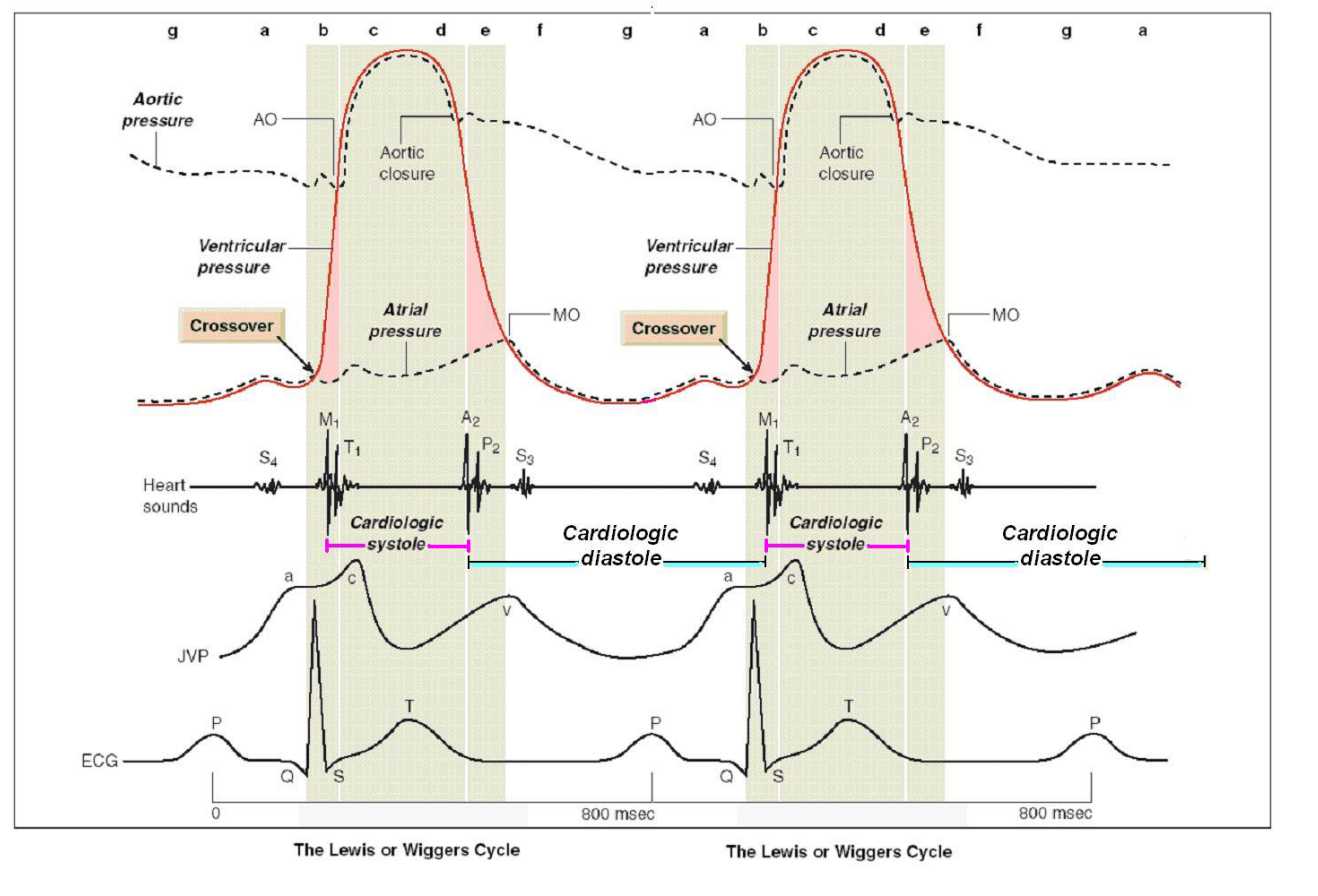
**The mechanical events in the cardiac cycle, first assembled by Lewis in 1920 but first conceived by Wiggers in 1915**. Cycle length of 800 milliseconds for 75 beats/min. *Cardiological systole *is demarcated by the interval between the first and the second heart sounds, lasting from the first heart sound to the closure of the aortic valve. The remainder of the cardiac cycle automatically becomes *cardiological diastole*. Left ventricular contraction: isovolumic contraction (b); maximal ejection (c). Let ventricular relaxation: start of relaxation and reduced ejection (d); isovolumic relaxation (e); LV filling rapid phase (f); slow LV filling (diastasis) (g); atrial systole or booster (a). Mitral valve closure occurs *after *the crossover point of atrial and ventricular pressures at the start of systole. A_2 _= aortic valve closure, aortic component of second sound; AO = aortic valve opening, normally inaudible; ECG = electrocardiogram; JVP = jugular venous pressure; M_1 _= mitral component of first sound at time of mitral valve closure; MO = mitral valve opening, may be audible in mitral stenosis as the opening snap; P_2 _= pulmonary component of second sound, pulmonary valve closure; S_3 _= third heart sound; S_4 _= fourth heart sound; T_1 _= tricuspid valve closure, second component of first heart sound. Modified from Opie LH. Mechanisms of cardiac contraction and relaxation. In: Braunwald E, Zipes DP, Libby P, Bonow RO, eds. Heart Disease. 7^th ^ed. WB Saunders Company 2005, Chap.19:457–489, page 475.

Physiologists and cardiologists are keenly aware of heart rate (HR) dependence of physiological indexes used to assess cardiac function. Selected pump-function indexes rely on stroke volume, typically based on systolic ejection, and significant work has been done to analyze the HR dependence of the duration of systole [1-6]. However, the assessment of the HR dependence of diastole and its phases has received little attention. Diastolic duration has previously been assessed as cardiac cycle duration (RR) minus electromechanical systole (QS_2_). Prior work concerning diastolic duration has been motivated primarily by consideration of diastolic myocardial perfusion time rather than the duration of mechanical events and has been used to assess the HR-dependent effects of pharmacological agents [7-9]. Recent studies utilizing both exercise radionuclide angiography time activity curve [10] or Doppler echocardiography [11,12] assessed that cardiac performance may be characterized in terms of the relative duration of systole and diastole. Cardiac cycle abnormalities of patients with heart failure are characterized by a prolongation of left ventricular systole and an abnormal shortening of left ventricular diastole. Reversal of the normal systolic/diastolic ratio may compromise cardiac filling and function. The systolic-diastolic mismatch is accentuated during exercise and has the potential to impair the cardiac reserve in these patients by restricting ventricular filling and perfusion. Ideally, the non-invasive, imaging independent, objective assessment of diastolic and systolic times at rest and during stress would greatly enhance its practical appeal. In Greek, *systole *means contraction and *diastole *means "to send apart". *Physiological systole *lasts from the start of isovolumic contraction to the peak of the ejection phase, so that physiological diastole commences as the LV pressure starts to fall. This concept fits well with the standard pressure-volume curve [13]. *Physiological diastole *commences as calcium ions are taken up into the SR, so that the myocyte relaxation dominates over contraction and the LV pressure starts to fall as shown on the pressure-volume curve. In contrast, *cardiological systole *is demarcated by the interval between the first and the second heart sounds, lasting from the first heart sound to the closure of the aortic valve. The remainder of the cardiac cycle automatically becomes *cardiological diastole *(Figure 1) (Table 1). Thus cardiological systole, demarcated by heart sounds rather than physiological events, includes: major part of the isovolumic contraction; maximal ejection; reduced ejection. Cardiological diastole includes second heart sound-first heart sound interval, filling phases included (Figure 1). Recently, a cutaneous operator independent force-frequency relation recording system has been validated in the stress echo lab, based on first heart sound amplitude variations at increasing heart rates [14-16]. Second heart sound can be easily simultaneously recorded by the same sensor [17-20]. Apart the first and the second heart sound vibrations amplitude (related to the isovolumic contraction force and to the isovolumic relaxation force) this new recording system could be utilized to automatically quantify both cardiological systole and diastole duration.

**Table 1:** Physiological versus cardiological systole and diastole

**Physiological systole**	**Cardiological systole**
Isovolumic contraction	From M_1 _to A_2_, including:
Maximal ejection	Major part of isovolumic contraction*
	Maximal ejection
	Reduced ejection
**Physiological diastole**	**Cardiological diastole**
Reduced ejection	A_2 _- M_1 _interval (filling phases included)
Isovolumic relaxation	
Filling phases	

* Note that M_1 _occurs with a definite albeit short delay after the start of the LV contraction. Modified from Opie LH. Mechanisms of cardiac contraction and relaxation. In: Braunwald E, Zipes DP, Libby P, Bonow RO, eds. Heart Disease. 7^th ^ed. WB Saunders Company 2005, Chap.19:457–489, page 474.

Aims of this study were:

1- To assess the feasibility of demarcate cardiological systole and cardiological diastole through an operator independent cutaneous heart sounds sensor.

2- To assess diastolic time at rest and at every 5 heart beats increase during exercise, dipyridamole or pacing stress in patients scheduled for stress echocardiography.

3- To assess systolic/diastolic time ratio at rest and at every 5 heart beats increase during stress.

4- To assess diastolic flow rate, at rest and at peak stress.

## Methods

### Patient selection

We enrolled 161 consecutive patients (106 males, 62 ± 13 years) referred for stress echocardiography (115 for exercise, 40 for dipyridamole, 6 for pacing stress). The type of stress was clinically driven for exercise vs. dipyridamole, and by the presence of a permanent pace maker for pacing stress. The characteristics of the study patients are reported in Table 2. The local Ethical Committee approved the study protocol. All of the patients gave their written informed consent before entering the study.

**Table 2:** Characteristics of the study patients

	**EXERCISE**	**DIP**	**PACING**
Pt n°	115	40	6
Age (years)	57 ± 14	66 ± 11	68 ± 10
Males	78	24	4
Controls	17	-	-
Previous PTCA/By pass	22	14	1
Previous myocardial infarction	24	10	2
Arterial hypertension	49	19	3
COPD	16	1	-
DC	17	1	1
Valvular disease	13	-	-
Atipical chest pain	7	9	3
BB on	39	21	4
ACEi on	32	9	3
Ca CB on	24	11	-

COPD = Chronic Obstructive Pulmonary Disease, DC = Dilated Cardiomyopathy, BB = Beta Blockers, ACEi = Angiotensin Converting Enzyme inhibitors, Ca CB = Calcium Channel Blockers

All patients met the following inclusion criteria: 1) referred to stress echo for clinically driven testing; 2) acoustic window of acceptable quality; 3) willingness to enter the study. Exclusion criteria were: 1) unstable angina or recent myocardial infarction; 2) moderate-to severe aortic stenosis; 3) hemodynamic instability, documentation of life-threatening ventricular arrhythmias (sustained ventricular tachycardia or ventricular fibrillation), atrial fibrillation.

From the initially considered population of 170 patients, 4 were excluded for poor acoustic window, 3 for low quality of the force sensor signal, or refusal to give written informed consent (n = 2).

### Stress echocardiography

#### Semi-supine bicycle exercise

Graded bicycle semi-supine exercise echo was performed in 115 patients starting at an initial workload of 25 watts lasting for 2 minutes; thereafter the workload was increased stepwise by 25 watts at 2 minutes interval. A 12-lead electrocardiogram and blood pressure determination were performed at baseline and every minute thereafter [21].

#### Dipyridamole stress echo

Dipyridamole stress echo was performed following the protocol of the American Society of Echocardiography [21,22], using Dipyridamole 0.84 mg/kg in 6' (accelerated protocol) in 40 patients. Contraindications of using dipyridamole were asthma, hypotension, and bradyarrhythmias.

#### Pacing stress echo

The study population consisted of 6 patients with a permanent Pace Maker. The pacing protocol was accelerated (with a 10-beat increment every 60 s). Stimulation was performed, wherever possible, in atrial stimulation mode, or dual-chamber (DDD) pacing to have normal contraction sequence. In the VVI-implanted patients, ventricular stimulation mode was used [23].

### Diagnostic end points and interruption criteria

The diagnostic end-points for all types of stress were: the development of obvious positive echocardiography, obvious alterations of ECG (ST segment shift > 3 mm). The exam was also stopped in case of limiting subjective side effects or hypertension (systolic pressure > 220 mmHg, diastolic pressure > 120 mmHg), hypotension (relative or absolute) with decrease of the blood pressure > 30 mmHg, supraventricular arrhythmias (supraventricular tachycardia, atrial fibrillation), ventricular arrhythmias (ventricular tachycardia, frequent and polymorphous ventricular beats); limiting dyspnoea, or maximal predicted heart rate in the absence of ischemia [21].

#### Regional wall motion analysis

The Wall Motion Score Index (WMSI) was calculated in each patient at baseline and peak stress, according to the recommendations of the American Society of Echocardiography from 1 = normal-hyperkinetic to 4 = dyskinetic in a 17 segment model of the left ventricle [21,24]. A test result was considered positive when the wall motion score increased by one grade or more at peak stress.

### Blood pressure analysis

One nurse recorded blood pressures both at rest and during each individual study. Blood pressure recording was made using a sphygmomanometer and the diaphragm of a standard stethoscope. Systolic and diastolic blood pressure was obtained on the right arm.

### Operator-independent cardiologic systole and diastole quantification

**Figure 2:**
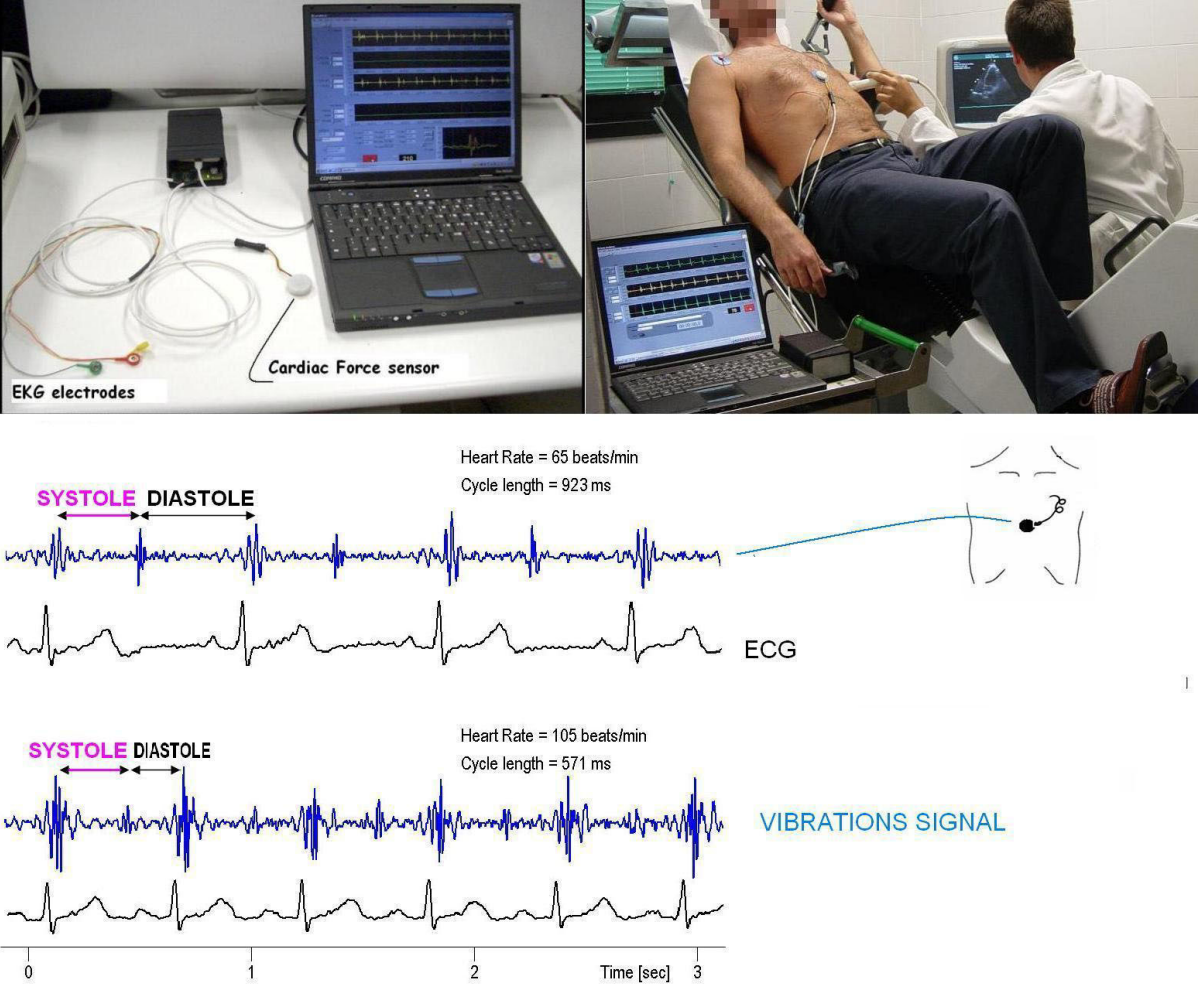
**Operator-independent cardiologic systole and diastole quantification**. The transcutaneous force sensor is based on a linear accelerometer from STMicroelectronics (LIS3). The device includes in one single package a MEMS sensor that measures a capacitance variation in response to movement or inclination and a factory trimmed interface chip that converts the capacitance variations into analog signal proportional to the motion. The device has a full scale of ± 2·g (g = 9.8 m/s^2^) with a resolution of 0.0005·g. We housed the device in a small case which was positioned in the mid-sternal precordial region and was fastened by a solid gel ECG electrode. The acceleration signal was converted to digital and recorded by a laptop PC, together with an ECG signal. The system is also provided with a user interface that shows both the acceleration and the ECG signals while the acquisition is in progress. The data were analyzed by using software developed in Matlab (The MathWorks, Inc). An analog peak-to-peak detector synchronized with the standards ECG scans the first 150 ms following the R wave to record first heart sound force vibrations and the 100 ms following the T wave to record second heart sound force vibrations. A stable, reproducible, and consistent first heart sound and second heart sound signal was obtained in all patients and utilized as time markers to continuously assess cardiologic systole and diastole during exercise, dipyridamole and pacing stress echo.

The transcutaneous force sensor is based on a linear accelerometer from STMicroelectronics (LIS3). The device includes in one single package a MEMS sensor that measures a capacitance variation in response to movement or inclination and a factory trimmed interface chip that converts the capacitance variations into analog signal proportional to the motion. The device has a full scale of ± 2·g (g = 9.8 m/s^2^) with a resolution of 0.0005·g. We housed the device in a small case (Figure 2) which was positioned in the mid-sternal precordial region and was fastened by a solid gel ECG electrode. The acceleration signal was converted to digital and recorded by a laptop PC, together with an ECG signal. The system is also provided with a user interface that shows both the acceleration and the ECG signals while the acquisition is in progress [15]. The data were analyzed by using software developed in Matlab (The MathWorks, Inc). An analog peak-to-peak detector synchronized with the standards ECG scans the first 150 ms following the R wave to record first heart sound force vibrations and the 100 ms following the T wave to record second heart sound force vibrations. The accelerometer simply records naturally generated heart vibrations, which audible components in the isovolumic (preejection) contraction period give rise to the first heart sound; while in the isovolumic relaxation period give rise to the second heart sound. Non myocardial noising vibrations (skeletal muscles, body movements, breathing) were eliminated by frequency filtering. Apart the first and the second heart sound amplitude (related to the isovolumic contraction force and to the isovolumic relaxation force) this recording system can be utilized to quantify both cardiological systole and diastole duration.

*According to the physiological background, cardiological systole *was demarcated by the interval between the first and the second heart sounds, lasting from the first heart sound to the closure of the aortic valve. The remainder of the cardiac cycle was automatically recorded as *cardiological diastole *(A_2 _- M_1 _interval, filling phases included) [13].

**Figure 3:**
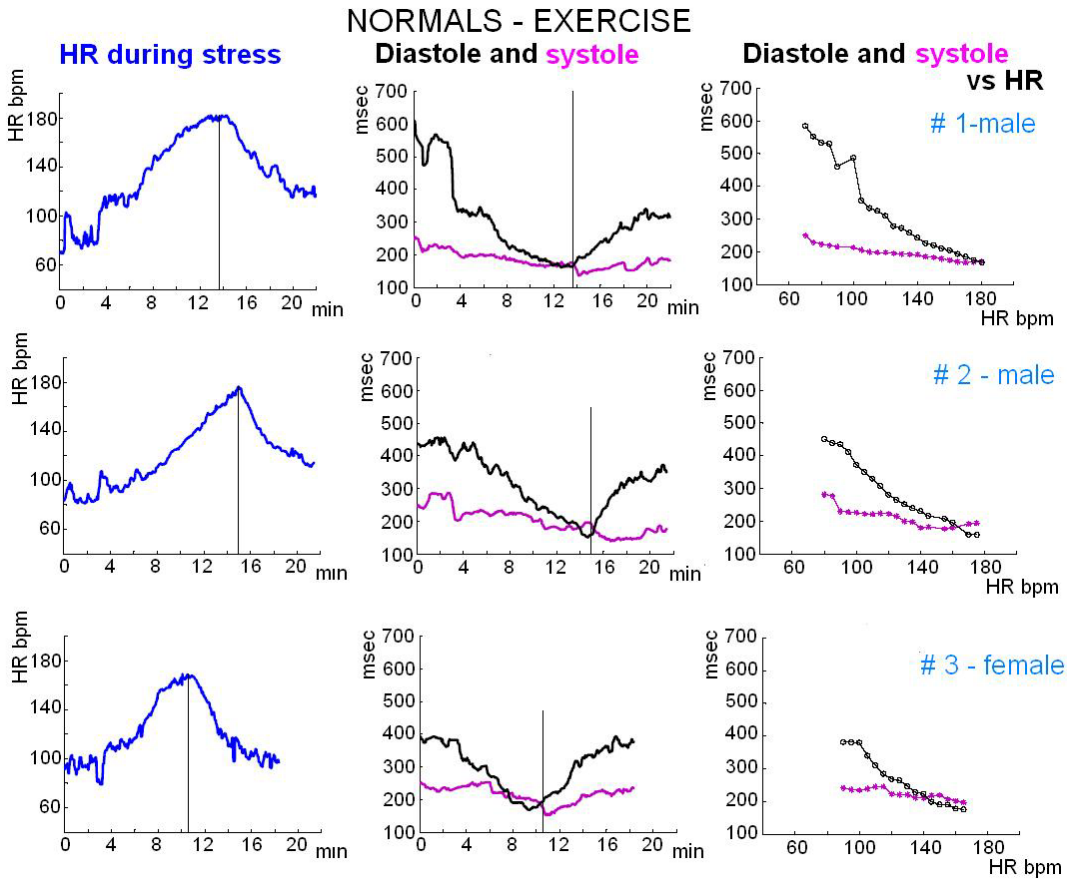
**Controls**. The curve of the systolic (pink line) and diastolic (black lines) time variation as a function of heart rate in 3 controls (upper and middle panels males, lower panel female). The parameters are acquired as instantaneous values at baseline and during stress. In normal healthy volunteer subjects performing bicycle exercise, the observed duration of diastole was greater than systole up to 160 beats per minute. The data can be also remotely read by a telemetric connection.

Systolic and diastolic times were acquired continuously at baseline and during stress. The primary outcome was the proportion of the cardiac cycle occupied by systole and diastole (absolute values, msec). Secondary outcomes included the systolic/diastolic ratio. The systolic/diastolic ratio was expressed as a dimensionless ratio (systolic/diastolic). The systolic and diastolic intervals were displayed by plotting time (y axis, msec) vs. heart rate increase during stress (x axis, heart rate, bpm) (Figure 3).

### Volume measurements and diastolic mitral filling rate quantification

*Left ventricular end-diastolic (EDV) and end-systolic (ESV) volumes *were measured from apical four- and two-chamber view at rest and at peak stress, by an experienced observer using the biplane Simpson-method [25]. Only representative cycles with optimal endocardial visualization were measured and the average of three measurements was taken. The endocardial border was traced, excluding the papillary muscles. The frame captured at the R wave of the ECG was considered to be the end-diastolic frame, and the frame with the smallest left ventricular cavity the end-systolic frame. Images were acquired at baseline and at peak stress.

*Stroke volume (SV) *was calculated at rest and at peak stress as EDV-ESV.

Because stroke volume varies directly with body size, it was indexed for body surface area (SVi) to better reflect differences with age and between the genders adjusted for differences in body size.

#### Cardiac index calculation

Cardiac index (SVi × heart rate) was calculated at baseline and peak stress.

#### Diastolic filling rate

Diastolic filling rate was calculated at rest and at peak stress as mitral filling volume (considered equivalent to the SVi estimated with biplane Simpson method) divided by diastolic time (automatically sensor estimated) × 1,000 [26,27].

### Intra- and inter-observer reproducibility

The intra- and inter-observer reproducibility was tested in 20 randomly selected patients at rest and during stress.

### Statistical analysis

SPSS 11 for Windows was utilized for statistical analysis. The statistical analyses included descriptive statistics (frequency and percentage of categorical variables and mean and standard deviation of continuous variables). The primary outcome was the proportion of the cardiac cycle occupied by systole and diastole (absolute value, msec). Secondary outcomes included the systolic/diastolic ratio. Repeated-measures analysis of variance (ANOVA) was used to compare data groups. The Scheffé test was used for post hoc comparisons. The correlation between the heart rate and the systolic, diastolic times, and the systolic/diastolic ratio was assessed by error bar charts plotting the standard deviations of individual variables at each 5 beats heart rate increase during stress (exercise, dip or pacing stress). Multiple linear regression analysis was used to analyze the data to allow for multiple parameters, including dependence on HR, subject-to-subject variability, and gender differences. The correlations between the diastolic duration time, the diastolic filling rate and the cardiac index were assessed by regression analysis and scatter plots, both at rest and at peak stress. A two-tailed *P*-value of < 0.05 was considered significant.

## Results

### Resting and stress echocardiographic findings

Technically adequate images were obtained in all patients at baseline (by selection) and during stress.

#### At Peak Exercise

Heart rate was lower in the dipyridamole than in the exercise and pacing groups. Regional wall motion abnormalities occurred in 2 patients of the exercise, 1 patient of Dip and 2 patients of the pacing groups. (Table 3)

**Table 3:** Rest and stress data

	**EXERCISE**		**DIP**		**PACING**
Pt n°	115		40		6
Age (years)	57 ± 14	Δ	66 ± 11		68 ± 10
Gender (M/F)	78/37		24/16		4/2
**Standard measurements**					
HR rest (bpm)	71 ± 14		66 ± 12		71 ± 10
HR peak (bpm)	128 ± 22	Δ	84 ± 14	*	132 ± 13
LV EF % rest	59 ± 12		61 ± 10		51 ± 11
WMSI rest	1.15 ± 0.33		1.10 ± 0.22		1.28 ± 0.46
WMSI peak	1.15 ± 0.34		1.11 ± 0.22		1.42 ± 0.46
SBP rest (mmHg)	132 ± 20		136 ± 22		133 ± 20
SBP peak (mmHg)	188 ± 24	§	130 ± 28		137 ± 37
DBP rest (mmHg)	73 ± 10		71 ± 13		74 ± 11
DBP peak (mmHg)	93 ± 12	§	68 ± 14		75 ± 15
**Sensor recorded times**					
Systolic time rest (msec)	315 ± 45	Δ	348 ± 36		315 ± 53
Systolic time peak (mesc)	236 ± 40	Δ	338 ± 43	*	249 ± 38
Diastolic time rest (msec)	541 ± 143	Δ	651 ± 146		600 ± 108
Diastolic time peak (msec)	250 ± 59	Δ	387 ± 98	*	211 ± 48
Systolic/diastolic time rest (ratio)	0.61 ± 0.14		0.56 ± 0.11		0.53 ± 0.08
Systolic/diastolic time peak (ratio)	0.98 ± 0.21	Δ	0.92 ± 0.20	*	1.25 ± 0.39

§= significant differences between exercise and both dipyridamole and pacing stress pts; * = significant differences between dipyridamole and pacing stress pts; Δ = significant differences between exercise and dipyridamole stress pts.

Despite similar baseline values, diastolic blood pressure increased in the exercise, decreased in the dipyridamole, while unchanged in the pacing group; although the response was heterogeneous at the individual level. (Table 3)

### Sensor diastolic and systolic times monitoring at increasing heart rates

**Figure 4:**
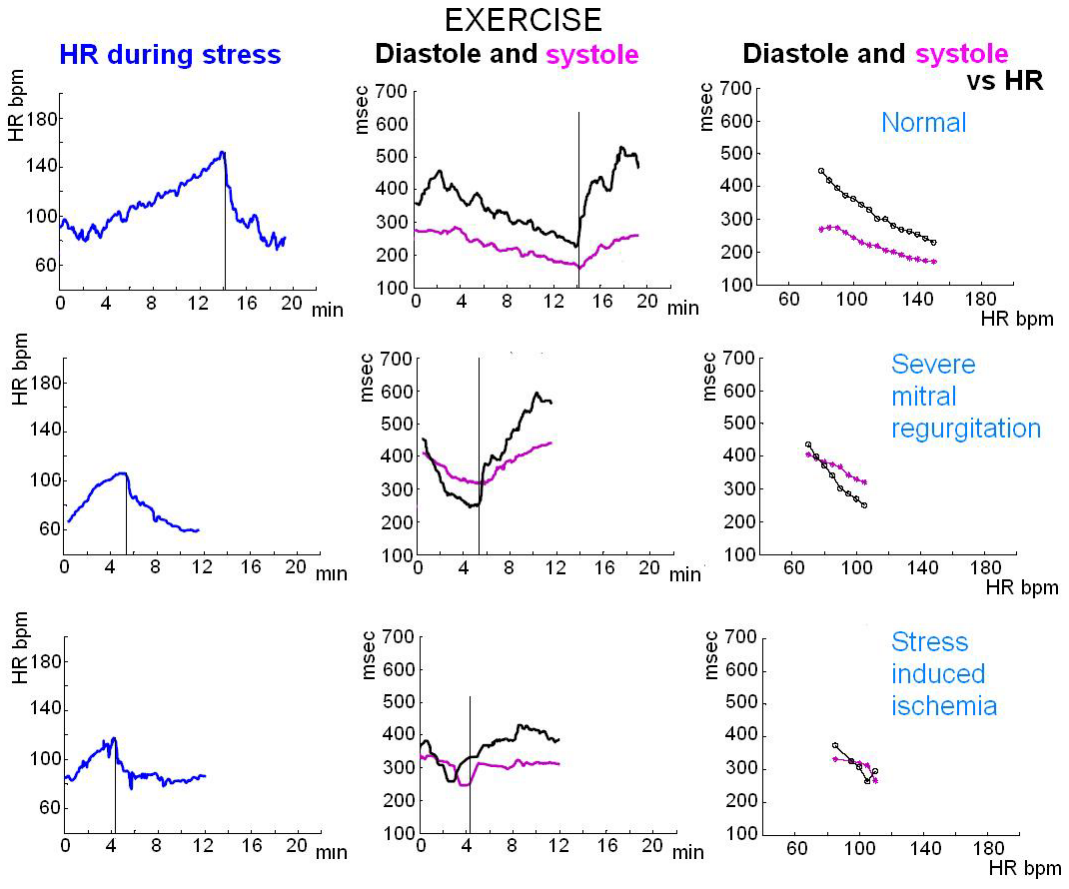
**Exercise stress echo**. Systolic (pink lines) and diastolic (black lines) times as a function of HR. Upper panel, a normal subject: systolic duration plotted as a function of HR demonstrates a slight linear decrease; duration of diastole demonstrates the most significant change. Middle panel, a patient with severe mitral regurgitation and stress induced severe pulmonary hypertension; the test was stopped at low stress load due to limiting dyspnoea without stress induced ischemia: prolonged systolic time with systolic/diastolic time reversal occurred during stress. Lower panel, a patient with stress induced ischemia at low stress load: at ischemia systole lengthens with systolic/diastolic time reversal.

**Figure 5:**
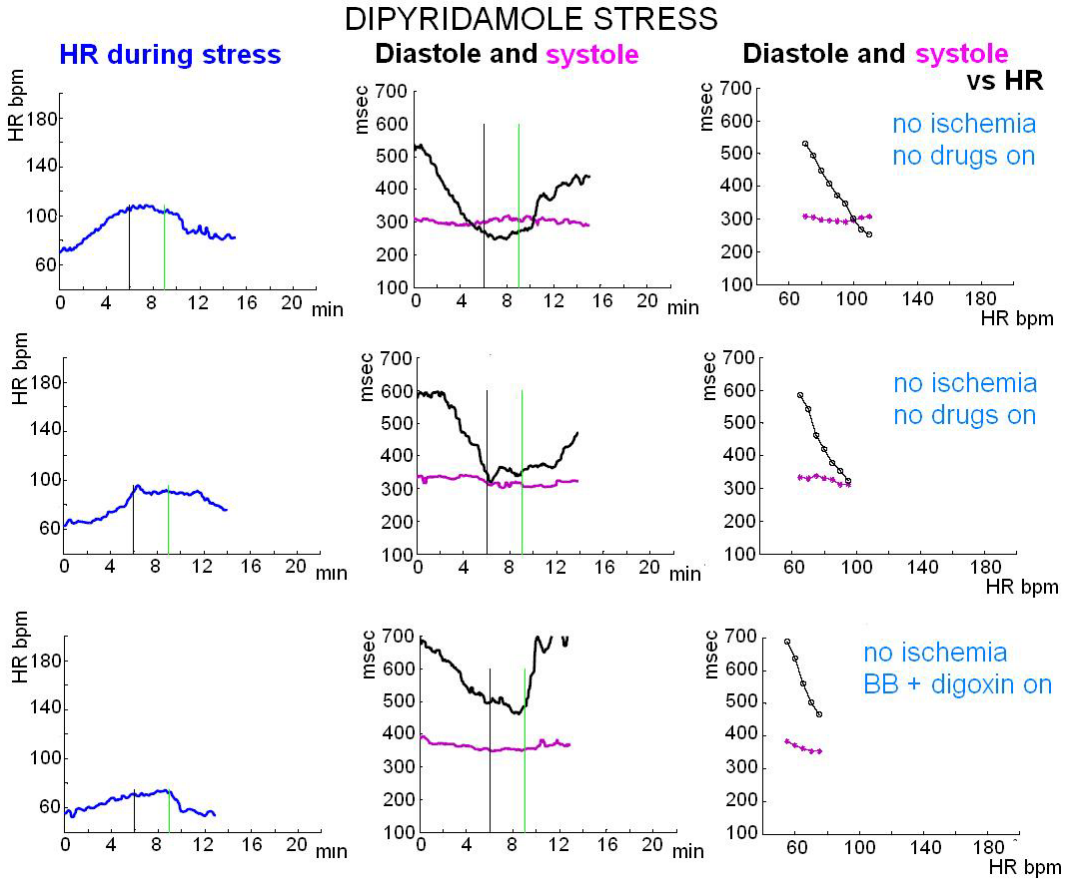
**Dipyridamole stress echo (0.84 mg/kg in 6', accelerated protocol)**. Systolic (pink lines) and diastolic (black lines) times as a function of HR. Dipyridamole results in adenosine receptor-mediated systemic as well coronary vasodilatation, often accompanied by a reflex increase in heart rate. With moderate stress induced HR increase (up to 110–100 bpm, upper and middle panel) diastolic time critically shortens, equalling systolic time. With mild induced HR increase (up to 70 bpm, lower panel) diastole has longer duration.

**Figure 6:**
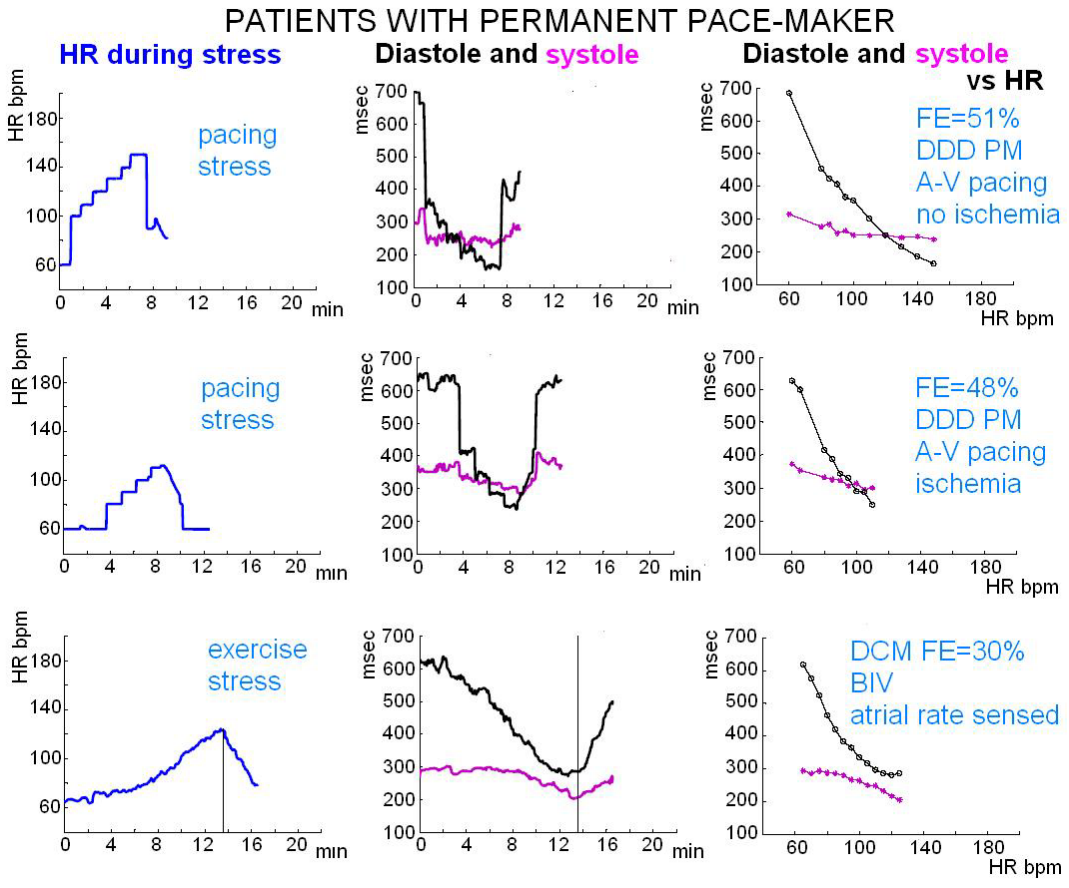
**Patients with a permanent Pace Maker**. Upper and middle panels, pacing stress echo: external programming of permanent PM induces a controlled change in heart rate which is independent of the patient capability to exercise; heart rate increase is achieved with sequential atrial-(right) ventricular stimulation with pacing induced left bundle branch block; a dramatic shortening of diastolic time occurs at pacing (from 60 bpm, spontaneous rhythmus to 100 bpm paced rhythmus) with a reversal of the systolic/diastolic ratio at 120 bpm heart rate). Lower panel, exercise stress in a patient with dilated cardiomyopathy (LVEF = 30%) and (sensed atrial rate) BIV pacing: the physiological induced increase in heart rate is accompanied by no reversal of the systolic/diastolic time ratio.

A consistent first heart sound and second heart sound signal was obtained in 161 out of 164 patients at rest and during stress. In 3 patients (2% of the examinations) data were discarded because of a low signal to noise ratio which was related to both small amplitude of the signal and the presence of several artefacts due to heavy movements and/or speaking of the patient. A typical systolic and diastolic times trend during exercise, dipyridamole and pacing stress is shown in Figure 4, 5 and 6.

**Figure 7:**
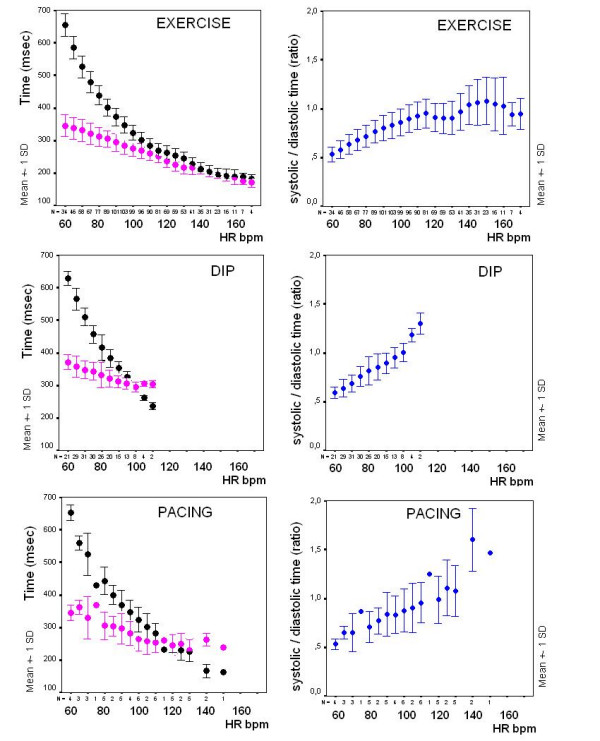
**Summary data**. Left panels: plot of systolic (pink symbols) and diastolic (black symbols) mean ± SD time values at increasing heart rates during exercise, dipyridamole and pacing stresses. Right panels: plot of systolic/diastolic time ratio as mean ± SD time values at increasing heart rates in the same patients groups; despite lower peak stress heart rates, dipyridamole patients show systolic/diastole time reversal at around 100 bpm heart rate.

Diastolic time decreased from 541 ± 143 to 250 ± 59 msec during exercise, from 651 ± 146 to 387 ± 98 msec in the dipyridamole group, and from 600 ± 108 to 211 ± 48 msec in the pacing group (Figure 7).

The systolic/diastolic time ratio increased from 0.64 ± 0.15 to 1 ± 0.23 in the exercise, from 0.57 ± 0.11 to 0.92 ± 0.19 in the dipyridamole group, and from 0.53 ± 0.08 to 1.25 ± 0.39 in the pacing group (Table 3) (Figure 7).

**Figure 8:**
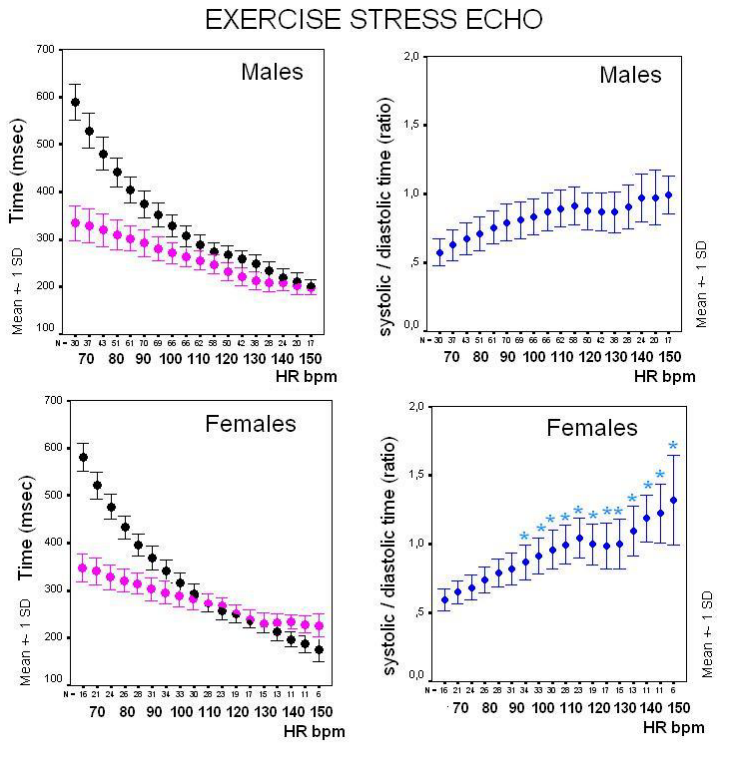
**Exercise stress, males vs. females**. Plot of systolic (pink symbols) and diastolic (black symbols) mean ± SD time values at increasing heart rates during exercise stress in males (upper panels) and females (lower panels). Despite similar prevalence of controls vs. heart diseases patients in both subgroups, females show lower diastole duration at peak stress.

In the exercise group as a whole, diastolic times were blunted in females vs. males at peak stress (Figure 8).

**Figure 9:**
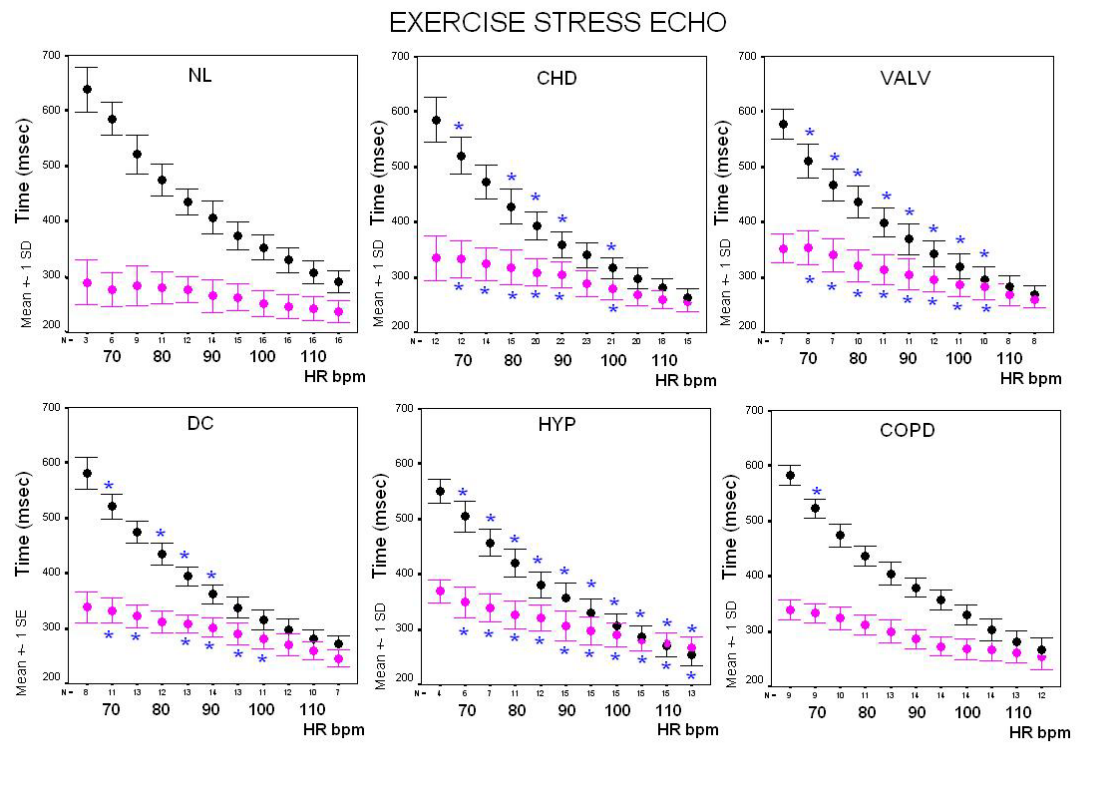
**Exercise stress, controls vs. patients**. Correlation between the heart rate and the systolic (pink symbols) and diastolic (black symbols) times is displayed by error bar charts plotting the standard deviations of individual variables at each 5 beats heart rate increase during stress. Repeated-measures analysis of variance (ANOVA) was used to compare data groups. At each stage of stress inter-group comparison was performed and significant differences (p < 0.05) are displayed with symbols: * = significant differences between controls (NL, left upper panel) and patients groups.

**Figure 10:**
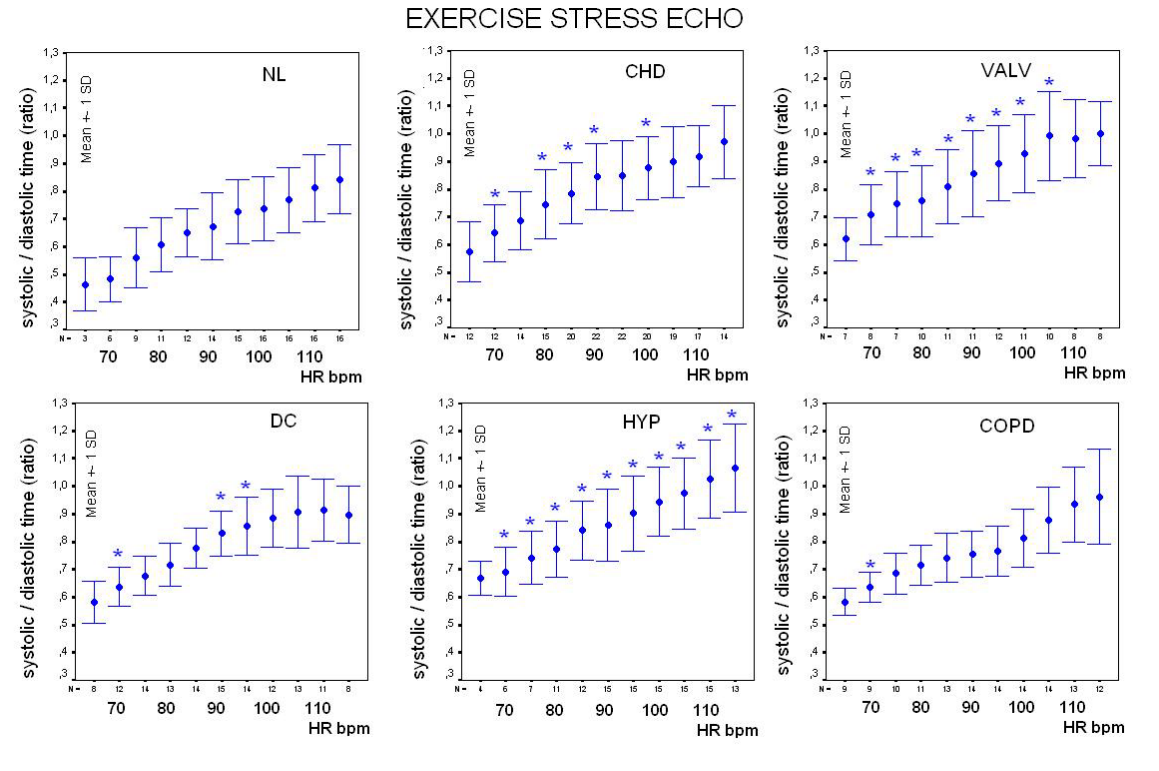
**Exercise stress, controls vs. patients**. Systolic/diastolic ratio plotted against heart rate in 17 controls (NL, left upper panel) and in patients groups during exercise stress. At each stage of exercise inter-group comparison was performed and significant differences (p < 0.05) are displayed with symbols: * = significant differences between controls and patients groups.

In the exercise group diastolic time was greater and systolic/diastolic time ratio lower in the 17 control subjects than in the 98 patients. At higher heart rates, diastole duration was blunted in patients with systemic hypertension, coronary, valvular or dilated heart disease. The relation between systole duration, diastole duration and the systolic/diastolic ratio to heart rate is demonstrated in Figure 9 and in Figure 10, showing the relative prolongation of systole and the elevated systolic/diastolic ratio in the patients at the expense of diastolic time and filling.

In the dipyridamole group (no controls), mean systolic and diastolic time occupied 35 ± 5% and 65 ± 5 % of the total cycle period, at rest. At drug infusion, the mild rise in heart rate shortened both portions of the cardiac cycle, but the decrease was greater with diastole. At peak stress, systolic time period was 47 ± 6 % of the cycle, while the estimated diastolic time was 53 ± 6 (Figure 7).

Individual's trends of 2 out of the 6 pacing stress are shown in Figure 6.

### Diastolic filling rate and cardiac index

By selection, mitral filling volume (= stroke volume index) was calculated in 102 out of the 161 patients. The diastolic filling rate was 101 ± 36 ml/m^2^* s^-1 ^at rest and increased significantly (*P *< 0.05) to 219 ± 92 ml/m^2^* s^-1 ^at peak stress in the whole group of patients.

**Figure 11:**
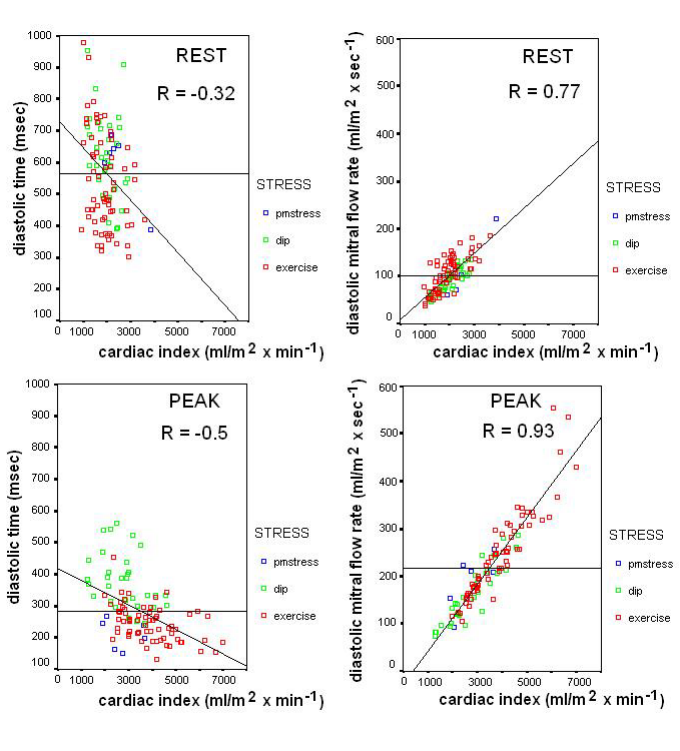
**Diastolic time and diastolic filling rate vs. cardiac index: summary data**. Left panels: scatter plots demonstrating relationships between the diastolic duration time and the cardiac index, both at rest and at peak stress. Right panels: scatter plots demonstrating relationships between the diastolic filling rate and the cardiac index, both at rest and at peak stress. Green symbols: dipyridamole stress; blue symbols: pacing stress; red symbols: exercise stress. The substantial increase in the LV diastolic filling rate measured as volume per time suggests a large filling capacity during exercise (red symbols).

Values for diastolic filling variables at rest and during exercise, dip and pacing stress are outlined in Table 4. Scatter plots demonstrating relations between the diastolic duration time, the diastolic filling rate and the cardiac index are shown in Figure 11.

**Table 4:** Mitral filling volume and diastolic filling rate

	**EXERCISE**		**DIP**		**PACING**
Pt n°	64		32		6
Age (years)	57 ± 13	Δ	66 ± 11		68 ± 10
Gender (M/F)	44/20		20/12		4/2
HR rest (bpm)	70 ± 14		67 ± 12		71 ± 10
HR peak (bpm)	129 ± 22	Δ	85 ± 13	*	132 ± 13
Diastolic time rest (msec)	531 ± 148	Δ	623 ± 135		600 ± 108
Diastolic time peak (msec)	245 ± 58	Δ	377 ± 87	*	211 ± 48
Mitral filling volume rest (= stroke volume index rest), ml/m^2^	28 ± 7		30 ± 7		35 ± 9
Mitral filling volume peak (= stroke volume index peak), ml/m^2^	30 ± 8	‡	33 ± 9	*	21 ± 6
diastolic filling rate rest (= mitral filling volume/diastolic time * 1,000), ml/m^2 ^* s ^-1^	105 ± 36		90 ± 26		117 ± 57
diastolic filling rate peak (= mitral filling volume/diastolic time*1,000), ml/m^2 ^* s ^-1^	248 ± 95	Δ	164 ± 60		191 ± 58
Cardiac index rest (ml/m^2 ^* min)	1915 ± 605		1987 ± 4922		2473 ± 709
Cardiac index peak (ml/m^2 ^* min)	3883 ± 1176	§	2783 ± 913		2745 ± 767

§= significant differences between exercise and both dipyridamole and pacing stress pts; ‡ = significant differences between exercise and pacing stress pts; * = significant differences between dipyridamole and pacing stress pts; Δ = significant differences between exercise and dipyridamole stress pts.

## Discussion

Recently, a cutaneous operator independent force-frequency relation recording system as been validated in the stress echo lab based on first heart sound vibrations amplitude variations at increasing heart rates [14,15]. Recording of the second heart sound vibrations can be easily simultaneously obtained in the same stress session. Cardiological systole, demarcated by heart sounds, includes: major part of the isovolumic contraction, maximal ejection; reduced ejection (Table 1 and Figure 1). Cardiological diastole includes second heart sound-first heart sound interval, filling phases included [13]. A stable, reproducible, and consistent first heart sound and second heart sound signal was obtained during stress. First heart sound vibrations are an expression of the tension wave produced during initial activation of the heart in the isovolumic contraction phase and are fairly consistent in sinus rhythm. Second heart sound vibrations generates from the abrupt deceleration of the moving aortic blood mass at the beginning of the isovolumic relaxation phase [17-20]. In this work the sensor monitored first and second heart sound vibrations were utilized as time markers to continuously assess cardiologic systole and diastole during exercise, dipyridamole and pacing stress echo, in a non-invasive, imaging independent, objective assessment manner. To our knowledge, there has been no prior study in humans that sought to characterize diastolic duration as a function of HR, with a non invasive, cutaneous sensor. Heart rate is the major determinant affecting diastole and systole duration. Systole is linearly related to heart rate, with the ejection time inversely related to heart rate. Diastole has a more complex relation with heart rate and is longer at low heart rates. Other Authors [12] with different methods, found that in normal, healthy volunteer performing supine bicycle exercise, the duration of diastasis, its shortening, and eventual disappearance as HR increases is the dominant factor accounting for the shortening of diastole duration.

In the whole group of patients, mean systolic and diastolic time occupied 37 ± 5% and 63 ± 5% of the cardiac cycle at rest.

The total cardiac cycle duration is algebraically dependent on the heart rate [= 60,000 msec/heart rate] with fixed values totally independent from the increasing heart rate stress type. (Table 5)

**Table 5:** Heart rate and total cardiac cycle duration

HR b.p.m	50	60	70	80	90	100	110	120	130	140	150	160	170	180
Cardiac cycle length (msec)	1200	1000	857	750	666	600	545	500	463	429	400	375	353	333

The total cardiac cycle duration is algebraically dependent on the heart rate [= 60,000 msec/heart rate] with fixed values totally independent from the increasing heart rate stress type.

However at each heart rate the fixed total cardiac cycle time can be differently divided between systole and diastole. At fixed heart rates the diastolic time fraction is determined by factors that modulates systolic duration through modulation of myocyte contraction [9]. Obviously, the method utilized to increase heart rate profoundly interacts with the adrenergic state of the heart and possibly modulating systolic time fraction. Exercise is accompanied by a two to tenfold increase of the plasma levels of norepinephrine. Dipyridamole results in adenosine receptor-mediated systemic as well coronary vasodilatation, often accompanied by a mild reflex increase in heart rate, without direct effects of the drug on diastolic time fraction [22]. Catecholamine levels are generally unchanged during the increase in heart rate produced by external programming of a permanent pace maker [23], but as for spontaneous LBBB, LV dyssynchrony induced by apical right ventricular pacing shortens diastolic time fraction at fixed heart rates. Cardiac resynchronization therapy is a great benefit to many patients with severe symptomatic heart failure, wide QRS duration, and depressed left ventricular ejection fraction, increasing systolic function and diastolic filling time as a ratio of cycle length.

In the exercise group of this study the systolic time declined as stress intensity increased, with a significant decrease at each level of exercise. From rest to peak exercise, the mean systolic time was shortened by 25 ± 11%. The diastolic time decreased markedly during early exercise and, at a heart rate of 100 bpm, the mean filling time had shortened by 35 ± 16% (by 52 ± 13% at peak stress). Others [28-30] have found similar systolic and diastolic times at high work intensities.

At 100 bpm, systolic/diastolic time ratio was lower in the 17 controls (0.74 ± 0.12) than in patients with hypertensive (0.94 ± 0.12), coronary (0.88 ± 0.11), valvular (0.93 ± 0.14) or cardiomyopathy heart (0.86 ± 0.10) disease (all p < 0.05 vs. controls) (Figure 9, Figure 10).

Reversal of the normal systolic/diastolic ratio may compromise cardiac filling and function. Stress-induced "systolic-diastolic mismatch" can be easily quantified by a disproportionate decrease of diastolic time fraction, and is associated to several cardiac diseases, possibly expanding the spectrum of information obtainable during stress.

In the dipyridamole group at rest, mean systolic and diastolic time occupied 36 ± 5% and 64 ± 5% of the total cycle period, respectively. It is not likely that dipyridamole by itself had an influence on diastolic time fraction. At six minutes drug infusion, the mild rise in heart rate shortened both portions of the cardiac cycle, but the decrease was greater with diastole. At peak exercise, systolic ejection period was 47 ± 6% of the cycle, while the estimated diastolic time was 53 ± 6%. Since shortening of diastole duration occurs greatly in the frequency ranges of dipyridamole infusion, with possible inversion of the normal systolic/diastolic time ratio, its knowledge could be useful in the evaluation of the coronary flow reserve [31].

### Clinical implications and different scenarios for the routine use of the sensor

#### Cardiovascular drugs and ischemia

Previous studies [3,7] demonstrated changes in diastolic time (calculated as the cycle length minus electromechanical systole – QS_2_) with various pharmacologic agents: propranolol increased diastole by slowing HR; dobutamine increased %diastolic time by shortening the QS_2_; cedilanid increased %diastolic time both by slowing the HR and shortening the QS_2_; isoproterenol increased HR and shortened the QS_2 _significantly. These data show that cardiovascular drugs may have significant effects on the relative duration of diastole either by affecting HR or the duration of systole. This may have clinical implications for patients with coronary artery disease and patients with left ventricular hypertrophy, since in both cases coronary flow in mostly diastolic. At a given coronary perfusion pressure, subendocardial perfusion is dependent on the ratio between the time the heart is in diastole and the duration of a complete heart cycle. The diastolic time fraction indicates the duration of absence of compression of intramural vessels during a heart beat and is used as input into theoretical models on coronary perfusion [9,31]. The absence of a unique relation between heart rate and diastolic time fraction on one hand and the dominant role of diastolic time fraction in subendocardial perfusion on the other hand also follow from the observation that, at the ischemic threshold, diastolic time fraction rather than heart rate correlates with the significance of coronary stenosis in patients. This indicates the relevance of monitoring diastole in the critically diseased heart. Although diastolic time fraction has been recognized as an important variable for subendocardial perfusion, diastolic time fraction has rarely been presented as an independent variable.

#### Diastolic function and diastolic filling rate

The filling rate, i.e. volume per time, was calculated by dividing the stroke volume index, by the filling time. The filling rate was 101 ± 36 ml/m^2 ^* s^-1 ^at rest and increased significantly (*p *< 0.05) to 219 ± 92 ml/m^2 ^* s^-1 ^at peak stress in the whole group of patients. In the exercise group, despite a substantial increase in heart rate and a reduction in filling time, the filling rate increased 3 times (to 281 ± 85 ml/m^2 ^* s^-1^) in controls and 2 times (to 239 ± 85 ml/m^2 ^* s^-1^) in patients from rest to peak exercise, suggesting a larger filling capacity in normal. These values are comparable with findings previously observed [[26,27,32] and [33]] in adults. The heart is considered to act as a forward or force pump, serving to satisfy the augmented circulatory needs of exercise. It has long been recognized, however, that the circulation of blood during exercise must involve a two-pump system, a central cardiac pump and a second peripheral pump, presumably multifactorial, which is responsible for returning blood to the heart [26]. In considering this dual system, it is obvious that the two pumps are interdependent and must, axiomatically, generate equivalent outputs. Given these observations, it is not unreasonable to suggest that determinants of peripheral pump function are as likely to serve as limiting factors to oxygen delivery as is the systolic function of the heart [34]. Indeed, the concept that the peripheral pump may play a key role in defining cardiac output and oxygen uptake is supported by the observation in some studies in endurance athletes [32,33] that the diastolic filling time of the heart at maximal exercise is approximately two thirds that of systolic ejection time. That is, the circulatory system may face a greater challenge in maintaining diastolic cardiac filling than in providing for systolic ejection.

Despite their potential importance, the components and characteristics of the peripheral pump are poorly understood, reflecting the technical difficulty in assessing variables that influence cardiac diastolic filling during exercise. Stress echocardiography is useful for the evaluation of patients with dyspnoea of possible cardiac origin and diastolic heart failure currently accounts for more than 50% of all heart failure patients. The diagnosis of heart failure with normal ejection fraction requires evidence of diastolic left ventricular dysfunction, obtained non-invasively by tissue Doppler (E/E' > 15). However, interpretable E/E' are not always obtained, since the most common source of uninterpretable tracings is fusion of E and A velocities due to tachycardia. A better (more feasible and sensitive) way to assess diastolic function during stress echo is needed. The combination of a cutaneous operator-independent force sensor and 3D stress echo allows a highly feasible, fast and informative assessment of mitral inflow rate, which could be impaired in presence of diastolic dysfunction and provide insight on a novel form – feasible at last! – of diastolic stress echocardiography.

## Limitations of the study

A potential limitation of this study is the lack of information regarding Doppler indexes or diastolic function or dysfunction during stress. Doppler echocardiography is the preferred method for non-invasive diastolic function assessment. Doppler-derived indexes have been used to characterize diastolic function in numerous cardiac disorders, including heart failure, myocardial infarction, hypertrophic cardiomyopathy, and hypertension. In current practice, most diastolic function indexes are derived by visual inspection of transmitral E- and A-waves. These shape-derived indexes include peak velocity of the E-wave (E_peak_), duration of the E-wave, acceleration (AT) and deceleration (DT) times of the E-wave, and area under the E-wave [velocity-time integral (VTI)]. Additional indexes include the peak velocity of the A wave (A_peak_) and the ratio of the E_peak _and A_peak _velocities (E/A) [35].

During exercise E- and A-waves become difficult to separate and discern when the A-wave merges with the E-wave and covers more than two-thirds of the E-wave deceleration, which typically occurs at heart rate > 100 beats/min [12]. Furthermore, details of E and A waves were not reliably discernable above 120 bpm due to noise and resolution limitations. Despite this uncertainty, it is often assumed that changes observed in the indexes represent changes in pathological processes [36]. However, studies have clearly established that traditional transmitral flow-derived diastolic indexes are load dependent, and, therefore, changes in DF indexes could be due to load variability rather than to pathology [37-42]. Newer load-independent Doppler-derived indexes of DF have been proposed, from velocity of propagation (*V*_p_), to annular velocities derived from Doppler tissue imaging (E'/A', E', E/E'), but there is no consensus as to whether these indexes are truly load independent [43-45].

It has been noted that E' is load independent in patients with chronic ischemic syndrome [46] but has shown variability with changing preload in normal subjects [41,47], in animals [44], and in patients undergoing haemodialysis [48]. Even E/E', which many studies find to be load independent [41,48,49], has proved in animal experiments to be preload dependent [44]. Despite studies that have shown DF indexes to be load dependent, no studies have derived diastolic indexes that compensate for load, continuously changing during stress [50].

Another potential limitation is the fact that cardiologic diastole includes filling phases, but also isovolumic relaxation [13]. Its inclusion may have a slight effect on our measured fitting parameters. The inclusion of isovolumic relaxation time, which is minimally HR dependent, would essentially add a constant (shift) to the data [12]. Because it is essentially a constant offset, it would not change the significant finding that it is the change in heart rate that modulates the normal or abnormal change in the duration of diastole.

Five (3 %) of the 161 patients had stress induced ischemia. The low rate of test positivity depends on many factors. The test indication class was not always I or IIa: low appropriateness in a high volume laboratory setting mainly depends on too often repeated tests in the absence clinical changes [51]. Second, stress test was often performed in young patients with low pre-test probability of CAD (17 controls and 19 patients with atypical chest pain). Third, 19 dilated cardiomyopathy patients, and 13 valvular disease patients were referred for stress echo to assess contractility reserve [52-54], 17 COPD patients to check pulmonary pressure and exercise tolerance. Fourth, 13 CAD patients underwent dipyridamole stress to assess coronary flow reserve.

## Conclusion

An operator independent cutaneous force sensor based on first heart and second heart sound vibrations amplitude recording may be utilized to automatically quantify cardiological systolic and diastolic duration.

A stable, reproducible, and consistent first heart sound and second heart sound signal has been obtained in all patients and utilized as a time markers to continuously assess cardiologic systole and diastole during exercise, dipyridamole and pacing stress echo. Diastolic time was greater and systolic/diastolic time lower in the 17 control subjects than in the patients. At higher heart rates, the increased systolic/diastolic ratio was accentuated in patients with systemic hypertension, or coronary, dilated or valvular heart disease, reflecting the relatively prolonged systole and shortened diastole in these patients.

Simultaneous calculation of stroke volume with echocardiography allowed quantifying diastolic filling rate and its increase with stress.

Portability of the sensor and simple remote transmission of the signal could allow telemonitoring in chronic heart failure patients.

## Competing interests

The authors declare that they have no competing interests.

## Authors' contributions

TB conceived this study, performed the data analysis, and drafted the manuscript; LV, CP, EPa, LP, DAR and MP were responsible for data collection and revised the manuscript; VG, EB, FF and MG were responsible for technology development and digital signal processing; GA gave a contribution to data discussion; EPi gave a contribution to the preparation of study design, data discussion, and critical revision of the manuscript. All authors read and approved the final manuscript.

## References

[B1] Akhras F, Rickards AF (1981). The relationship between QT interval and heart rate during physiological exercise and pacing. Jpn Heart J.

[B2] Bazett HC An analysis of the time-relations of electrocardiograms. Heart.

[B3] Boudoulas H, Gleris P, Lewis RP, Rittgens SE (1981). Linear relationship between electrical systole, mechanical systole and heart rate. Chest.

[B4] Kovács SJ (1985). The duration of the QT interval as a function of heart rate: A derivation based on physical principles and a comparison to measured values. Am Heart J.

[B5] Taran LM, Szilagyi N (1947). The duration of the electrical systole (Q-T) in acute rheumatic carditis in children. Am Heart J.

[B6] Weissler AM, Harris WS, Schoenfeld CD (1968). Systolic time intervals in heart failure in man. Circulation.

[B7] Boudoulas H, Rittgers L, Leier CV, Weissler AM (1979). Changes in diastolic time with various pharmacologic agents: implication for myocardial perfusion. Circulation.

[B8] Meiler SEL, Bouldoulas H, Unverferth DV, Leier CV (1987). Diastolic time in congestive heart failure. Am Heart J.

[B9] Merkus D, Kajiya F, Vink H, Vergroesen I, Dankelman J, Goto M, Spaan JAE (1999). Prolonged diastolic time fraction protects myocardial perfusion when coronary blood flow is reduced. Circulation.

[B10] Plehn G, Vormbrock J, Zuhlke C, Christ M, Perings C, Perings S, Trappe HJ, Meissner A (2007). Disproportionate shortening of left ventricular diastolic duration in patients with dilated cardiomyopthy. Med Klin.

[B11] Friedberg MK, Silverman NH (2006). Cardiac ventricular diastolic and systolic duration in children with heart failure secondary to idiopathic dilated cardiomyopathy. Am J Cardiol.

[B12] Chung S, Karamanoglu M, Kovács SJ (2004). Duration of diastole and its phases as a function of heart rate during supine bicycle exercise. Am J Physiol Heart Circ Physiol.

[B13] Opie LH, Braunwald E, Zipes DP, Libby P, Bonow RO (2005). Mechanisms of cardiac contraction and relaxation. Heart Disease.

[B14] Gemignani V, Bianchini E, Faita F, Giannoni M, Pasanini E, Picano E, Bombardini T (2007). Operator independent force-frequency relation monitoring during stress with a new transcutaneous cardiac force sensor. Proc 34th Annual Conference of Computers in Cardiology.

[B15] Bombardini T, Gemignani V, Bianchini E, Venneri L, Petersen C, Pasanisi E, Pratali L, Pianelli M, Faita F, Giannoni M, Picano E, Cardiac reflections and natural vibrations Force-frequency relation recording system in the stress echo lab. Cardiovasc Ultrasound.

[B16] Bombardini T (2005). Method and device for the diagnosis and therapy of chronic heart failure. United States Patent.

[B17] Sabbah H, Stein P (1976). Investigation of the theory and mechanism of the origin of the second heart sound. Circ Res.

[B18] Vermarien H, Ghista DN, Ain A (1989). Mapping and vector analysis of heart vibration data obtained by multisite phono-cardiography. Advances in Cardiovascular Physics.

[B19] Stein P, Sabbah H, Anbe T, Khaja F (1978). Hemodynamic and anatomic determinants of relative differences in amplitude of the aortic and pulmonary components of the second heart sound. Am J Cardiol.

[B20] Stein P, Sabbah H, Khaja F, Anbe T (1978). Exploration of the cause of low intensity aortic component of the second sound in non hypotensive patients with poor ventricular performance. Circulation.

[B21] Armstrong WF, Pellikka PA, Ryan T, Crouse L, Zoghbi WA (1998). Stress echocardiography: recommendations for performance and interpretations of stress echocardiography. Stress Echocardiography Task Force of the Nomenclature and Standards Committee of the American Society of Echocardiography. J Am Soc Echocardiogr.

[B22] Picano E (1992). Stress echocardiography. From pathophysiological toy to diagnostic tool. Circulation.

[B23] Bombardini T, Agrusta M, Natsvlishvili N, Solimene F, Pap R, Coltorti F, Varga A, Mottola G, Picano E (2005). Noninvasive assessment of left ventricular contractility by pacemaker stress echocardiography. Eur J Heart Failure.

[B24] Cerqueira MD, Weissman NJ, Dilsizian V, Jacobs AK, Kaul S, Laskey WK, Pennell DJ, Rumberger JA, Ryan T, Verani MS, American Heart Association Writing Group on Myocardial Segmentation and Registration for Cardiac Imaging (2002). Standardized myocardial segmentation and nomenclature for tomographic imaging of the heart: a statement for healthcare professionals from the Cardiac Imaging Committee of the Council on Clinical Cardiology of the American Heart Association. Circulation.

[B25] Schiller NB, Shah PM, Crawford M, De Maria A, Devereux R, Feigenbaum H, Gutgesell H, Reichek N, Sahan D, Schnittger I (1989). Recommendations for quantitation of the left ventricle by two-dimensional echocardiography. American Society of Echocardiography Committee on Standards, Subcommittee on Quantification of Two-Dimensional Echocardiograms. J Am Soc Echocardiogr.

[B26] Rowland T, Mannie E, Gawle L (2001). Dynamics of left ventricular diastolic filling during exercise. A Doppler echocardiographic study of boys 10 to 14 years old. Chest.

[B27] Sundstedt M, Hedberg P, Jonason T, Ringqvist I, Henriksen E (2007). Echocardiographic Doppler assessments of left ventricular filling and ejection during upright exercise in endurance athletes. Clin Physiol Funct Imaging.

[B28] Cumming GR, Edwards AH (1962). Indirect measurement of left ventricular function during exercise. Can Med Assoc J.

[B29] Turkevich D, Micco A, Reeves JT (1988). Noninvasive measurement of the decrease in left ventricular filling time during maximal exercise in normal subjects. Am J Cardiol.

[B30] Van Fraechen JHP (1979). Stroke volume and systolic time interval adjustments during bicycle exercise. J Appl Physiol.

[B31] Fokkema DS, VanTeeffelen J, Dekker S, Vergroesen I, Reitsma JB, Spaan J (2005). Diastolic time fraction as a determinant of subendocardial perfusion. Am J Physiol Heart Circ Physiol.

[B32] Gledhill N, Cox D, Jamnik R (1994). Endurance athletes' stroke volume does not plateau: major advantage is diastolic function. Med Sci Sports Exerc.

[B33] Higginbotham MB, Morris KG, Williams RS, McHale PA, Coleman RE, Cobb FR (1986). Regulation of stroke volume during submaximal and maximal upright exercise in normal man. Circ Res.

[B34] Libonati JR (1999). Myocardial diastolic function and exercise. Med Sci Sports Exerc.

[B35] Galderisi M (2005). Diastolic dysfunction and diastolic heart failure: diagnostic, prognostic and therapeutic aspects. Cardiovasc Ultrasound.

[B36] Shmuylovich L, Kovács SJ (2006). Load-independent index of diastolic filling: model-based derivation with in vivo validation in control and diastolic dysfunction subjects. J Appl Physiol.

[B37] Downes TR, Nomeir AM, Stewart K, Mumma M, Kerensky R, Little WC (1990). Effect of alteration in loading conditions on both normal and abnormal patterns of left ventricular filling in healthy individuals. Am J Cardiol.

[B38] Choong CY, Herrmann HC, Weyman AE, Fifer MA (1987). Preload dependence of Doppler-derived indexes of left ventricular diastolic function in humans. J Am Coll Cardiol.

[B39] Hurrell DG, Nishimura RA, Ilstrup DM, Appleton CP (1997). Utility of preload alteration in assessment of left ventricular filling pressure by Doppler echocardiography: a simultaneous catheterization and Doppler echocardiographic study. J Am Coll Cardiol.

[B40] Masuyama T, St Goar FG, Alderman EL, Popp RL (1990). Effects of nitroprusside on transmitral flow velocity patterns in extreme heart failure: a combined hemodynamic and Doppler echocardiographic study of varying loading conditions. J Am Coll Cardiol.

[B41] Paelinck BP, van Eck JWM, De Hert SG, Gillebert TC (2003). Effects of postural changes on cardiac function in healthy subjects. Eur J Echocardiogr.

[B42] Stoddard MF, Pearson AC, Kern MJ, Ratcliff J, Mrosek DG, Labovitz AJ (1989). Influence of alteration in preload on the pattern of left ventricular diastolic filling as assessed by Doppler echocardiography in humans. Circulation.

[B43] Garcia MJ, Smedira NG, Greenberg NL, Main M, Firstenberg MS, Odabashian J, Thomas JD (2000). Color M-mode Doppler flow propagation velocity is a preload insensitive index of left ventricular relaxation: animal and human validation. J Am Coll Cardiol.

[B44] Jacques DC, Pinsky MR, Severyn D, Gorcsan J (2004). Influence of alterations in loading on mitral annular velocity by tissue doppler echocardiography and its associated ability to predict filling pressures. Chest.

[B45] Nagueh SF, Lakkis NM, Middleton KJ, Spencer WH, Zoghbi WA, Quinones MA (2003). Doppler estimation of left ventricular filling pressures in patients with hypertrophic cardiomyopathy. Circulation.

[B46] Yalcin F, Kaftan A, Muderrisoglu H, Korkmaz ME, Flachskampf F, Garcia M, Thomas JD (2002). Is Doppler tissue velocity during early left ventricular filling preload independent?. Heart.

[B47] Pela G, Regolisti G, Coghi P, Cabassi A, Basile A, Cavatorta A, Manca C, Borghetti A (2004). Effects of the reduction of preload on left and right ventricular myocardial velocities analyzed by Doppler tissue echocardiography in healthy subjects. Eur J Echocardiogr.

[B48] Oguzhan A, Arinc H, Abaci A, Topsakal R, Eryol NK, Ozdogru I, Basar E, Ergin A (2005). Preload dependence of Doppler tissue imaging derived indexes of left ventricular diastolic function. Echocardiography.

[B49] Lisauskas JB, Singh J, Bowman AW, Kovács SJ (2001). Chamber properties from transmitral flow: prediction of average and passive left ventricular diastolic stiffness. J Appl Physiol.

[B50] Bombardini T, Nevola E, Giorgetti A, Landi P, Picano E, Neglia D (2008). The prognostic value of left ventricular and peripheral vascular performance in patients with dilated cardiomyopathy. Journal of Nuclear Cardiology.

[B51] Bombardini T, Galderisi M, Agricola E, Coppola V, Mottola G, Picano E Negative stress echo: Further prognostic stratification with assessment of pressure-volume relation. Int J Cardiol.

[B52] Bombardini T Myocardial contractility in the echo lab: molecular, cellular and pathophysiological basis. Cardiovasc Ultrasound.

[B53] Bombardini T, Correia MJ, Cicerone C, Agricola E, Ripoli A, Picano E (2003). Force-frequency relationship in the echocardiography laboratory: a noninvasive assessment of Bowditch Treppe?. J Am Soc Echocardiogr.

[B54] Agricola E, Bombardini T, Oppizzi M, Margonato A, Pisani M, Melisurgo G, Picano E (2005). Usefulness of latent left ventricular dysfunction assessed by Bowditch Treppe to predict stress induced pulmonary hypertension in minimally symptomatic severe mitral regurgitation secondary to mitral valve prolapse. Am J Cardiol.

